# Hyaluronidase Modulates Inflammatory Response and Accelerates the Cutaneous Wound Healing

**DOI:** 10.1371/journal.pone.0112297

**Published:** 2014-11-13

**Authors:** Marcio Fronza, Guilherme F. Caetano, Marcel N. Leite, Claudia S. Bitencourt, Francisco W. G. Paula-Silva, Thiago A. M. Andrade, Marco A. C. Frade, Irmgard Merfort, Lúcia H. Faccioli

**Affiliations:** 1 Departamento de Análises Clínicas, Toxicológicas e Bromatológicas, Faculdade de Ciências Farmacêuticas de Ribeirão Preto, Universidade de São Paulo, Ribeirão Preto, São Paulo, Brazil; 2 Departamento de Clínica Médica, Divisão de Dermatologia, Faculdade de Medicina de Ribeirão Preto, Universidade de São Paulo, Ribeirão Preto, São Paulo, Brazil; 3 Department of Pharmaceutical Biology and Biotechnology, University of Freiburg, Freiburg, Germany; 4 Departamento de Farmácia, Universidade de Vila Velha, Vila Velha, Espirito Santo, Brazil; Institute for Frontier Medical Sciences, Kyoto University, Japan

## Abstract

Hyaluronidases are enzymes that degrade hyaluronan an important constituent of the extracellular matrix. They have been used as a spreading agent, improving the absorption of drugs and facilitating the subcutaneous infusion of fluids. Here, we investigated the influence of bovine testes hyaluronidase (HYAL) during cutaneous wound healing in *in vitro* and *in vivo* assays. We demonstrated in the wound scratch assay that HYAL increased the migration and proliferation of fibroblasts *in vitro* at low concentration, e.g. 0.1 U HYAL enhanced the cell number by 20%. HYAL presented faster and higher reepithelialization in *in vivo* full-thickness excisional wounds generated on adult Wistar rats back skin already in the early phase at 2^nd^ day post operatory compared to vehicle-control group. Wound closured area observed in the 16 U and 32 U HYAL treated rats reached 38% and 46% compared to 19% in the controls, respectively. Histological and biochemical analyses supported the clinical observations and showed that HYAL treated wounds exhibited increased granulation tissue, diminished edema formation and regulated the inflammatory response by modulating the release of pro and anti-inflammatory cytokines, growth factor and eicosanoids mediators. Moreover, HYAL increased gene expression of peroxisome proliferator-activated receptors (PPAR) γ and PPAR β/δ, the collagen content in the early stages of healing processes as well as angiogenesis. Altogether these data revealed that HYAL accelerates wound healing processes and might be beneficial for treating wound disorders.

## Introduction

A wound can be defined as a disruption of the anatomical, normal cellular and functional continuity of a structure. Thus, wound healing is a succession of complicated biochemical and cellular events that aims to restore the structural and functional integrity of the wounded tissue. The healing of cutaneous wounds is a multifaceted biological process that can be divided in three overlapping phases: inflammation, tissue formation, and tissue remodeling. The extracellular matrix synthesis and remodeling occurs during the entire processes [1], [2]. Immediately after the skin injury, a rapid and coordinated response of several cell types is triggered, including circulating platelets, leukocytes, keratinocytes, fibroblasts and endothelial cells. The release of cytokines, eicosanoids, growth factors and activation of transcriptional regulation genes are required to mediate the communication between different cell types aiming the proper healing of full-thickness wounds [3]–[5]. Defects or imbalance in this process might destroy the delicate equilibrium of cells and soluble factors necessary for complete wound repair, resulting in fibrotic scar [6], [7]. These complex interaction processes responsible for the homeostasis of mature skin is tightly regulated through different molecular targets. Among such targets are transcription factors that control various pathways in cellular repair. In particular, the involvement of peroxisome proliferator-activated receptors (PPARs) has received special attention for their protective and healing attributes in tissue injury and wound repair [8], [9].

Hyaluronic acid (HA) is a structural component of the extracellular matrix (ECM), but can also be located intracellular and increased following trauma. In the earliest phase of wound healing, there is a strict increase in HA in the site of injury, as a result of a combination of increased synthesis and impaired clearance [10], [11]. The role of HA during wound repair is not well understood. HA displays antioxidant properties and can modulate wound healing by promoting cell migration and proliferation, facilitating leukocytes infiltration and improving tissue hydration [12], [13]. However, elevated HA levels are often observed in hyperproliferative epidermis in the setting of acute inflammation, so-called inflammatory hyperplasia [14]. Degradation of HA mostly results from the enzymatic action of HYAL [15] which was recognized as a “spreading factor” by hydrolyzing the dermal barrier. HYAL has been therapeutically used for many years based on their properties in facilitating the subcutaneous infusion and dispersion of fluids thus improving absorption of drugs [16]–[19], besides its ability to reduce bleomycin-induced lung injury and fibrosis [20]–[22].

Although HYAL is involved in wound healing the exact mode of action is still unknown. Therefore, this study propose to investigate the mechanisms of action of the bovine testes HYAL in different stages of cutaneous wound healing using *in vitro* and *in vivo* models. Our results demonstrated that HYAL accelerated the wound healing process and might be beneficial for treating wound disorders.

## Materials and Methods

### Cell lines, chemicals and biochemicals

Swiss 3T3 albino mouse fibroblasts (Cell Line Service, Rio de Janeiro, Brazil - ATCC CCL-92) were maintained in Dulbecco's modified Eagle's medium (DMEM) supplemented with 10% fetal bovine serum (FBS), 100 IU/ml penicillin and 100 µg/ml streptomycin, at 37°C in a containing 5% CO_2_ humidified atmosphere (all Gibco-BRL, Netherlands). HYAL from bovine testes (H3884), collagen solution, type I from rat tail and platelet derived growth factor-BB (PDGF) were from Sigma Chemical Co, MO, USA, Mitomycin C (Bristol-Myers) from a local pharmacy shop, enzyme-linked immunosorbent assays (ELISAs) for TNF-α, IFN-γ, IL-6, IL1-α, IL1-β, IL-10, IL-4, IL-5, TGF-β1 and VEGF from R & D Systems (Minneapolis, USA) and for the eicosanoids (PGE_2_, PGD_2_ and LTB_4_) from Cayman Chemical (An Arbor, USA). Coomassie protein assay reagent from Thermo Scientific (Rockford, USA). Illustra RNAspin Mini Isolation Kit from GE Healthcare, (UK). Reverse transcription (High Quality cDNA Reverse Transcriptase Kit) and TaqMan primers were from Applied Biosystems (CA, USA).

### Animals

48 adult male Wistar rats (*Rattus norvegicus*) (180–230 g), aged 6 to 7 weeks were obtained from the central Bioterium of the Medical School, Ribeirão Preto, University of São Paulo (FMRP-USP). In order to prevent skin lesions from fighting males, which may interfere with wound repair, animals were housed singly (1 week pre- and for the entire period post wounding). Animals were maintained under standard laboratory conditions with a 12 h light-dark cycle and a free access to food and water. All mouse experiments were conducted in accordance with the Brazilian Committee for animal care and use (COBEA) guidelines and approved by the University Animal Care Committee at USP-RP (process 2012.1.397.53.2).

### *In vitro* cell migration assay

The proliferation and migration abilities of fibroblasts exposed to hyaluronidase were assessed using a scratch wound assay, which measures the expansion of a cell population on surfaces. The assay was performed as previously described [23] and in the Protocol S1.

### *In vivo* wound-healing experiments

The animals were randomly divided in three groups (n = 16) and subdivided in four subgroups (n = 4). The groups were evaluated for 21 days in defined post-operatory periods according to standards protocols [24], [25]. The following groups were used: in the vehicle-control group (control) the animals were only treated with 0.2 g/wound of 2% hydroxyethylcellulose gel base; in the hyaluronidase 16 U (HYAL 16 U) and hyaluronidase 32 U (HYAL 32 U) groups the wounds were treated with hydroxyethylcellulose gel in a way that 0.2 g contained 16 and/or 32 U of active drug/wound, respectively. The pH value in the gels was monitored to be around 6.0 confirming optimal conditions for bovine testis HYAL activity [26]. The enzymatic activity of the HYAL used in the current study was turbidimetrically determined before and after the gel preparations using the methodology described by Pessini et al. (2001) [27]. Hydrolytic activity of hyaluronidase was preserved in the gel preparation (Table S1). Detailed information is given in the Protocol S1. The choice of hydroxyethylcellulose gel delivery system was based on its wide spread application in the pharmaceutical industry and its high biocompatibility [28]. As a comparator arm, a vehicle control containing the same formulation as the study product without the active agent was used [29]. Prior to the excisional wound induction, the animals were weight and deeply anesthetized by intraperitoneal (i.p.) administration of an association between ketamin (80 mg/Kg) and xylazine (15 mg/Kg). After shaving and cleaning with 70% ethanol two full thickness excision wounds were made on the dorsum cervical region of each rat with sterile 150 mm punch biopsy (Stiefel Laboratories, Offenbach, Germany). Immediately after the surgery and daily afterwards on the same hour, the control group and the HYAL treated groups received their respective treatments. The animals were euthanatized at 2^nd^, 7^th^, 14^th^ and 21^st^ post-operatory days and the wounds and their surrounding areas were cut with a sterile biopsy punch. One wound of each animal was snap frozen in liquid nitrogen and stored at −70°C. The other wound was used to perform histological analysis.

### Rate of wound closure determination – morphometric evaluation

The morphometric analysis of the wounds was performed using images of the wounds at days zero, 2, 5, 7, 10, 14 and day 21 post-operatory to determine the remained wound area using Image J software (NIH, USA) [30]. The rate of wound closure that represents the percentage of wound reduction from the original wound size was calculated using the following formula: wound area day 0 – wound area (day 2, 5, 7, 10, 14 and 21)/wound area day 0×100. Values expressed as percentage of the healed wounds.

### Histological analysis

The wound specimens were fixed in 4% phosphate-buffered formaldehyde at days 0, 2, 7, 14 and 21 post-wounding and processed according to the standard routine light microscope tissue protocols. The processed tissues were embedded in paraffin and serial sections of 5 µm-thick were mounted on glass slides, dewaxed, rehydrated to distilled water, and stained with hematoxylin and eosin (H&E) as well as with the solution of Sirius Red F3BA saturated in aqueous picric acid [20]. The slides were examined and photomicrographed in a blinded fashion using digital camera (LEICA DFC 280, Germany) attached to a light microscope (LEICA DM 4000B, Germany). Quantitative analysis of inflammatory infiltrate and angiogenesis by image analysis was determined as previously described [24], [31] and in the Protocol S1. Photographs taken from the Picrosirius red-stained sections were used for quantifying the collagen content in the wound tissue [24]. The morphometric analysis corresponding to the area occupied by the fibers were determined by digital densitometry recognition and expressed as percentage of the total area of the field using Image J software (NIH, USA). Detailed information is given in the Protocol S1.

### Cytokines and lipid mediators measurements

Tissue sections of wound biopsies treated with vehicle-control or HYAL 16 U were homogenized (Mixer Homogenizer, Labortechnik, Germany), centrifuged at 1500 g and stored at −70°C until assayed. The homogenate fluid obtained were used to measure TNF-α, IFN-γ, IL-6, IL1-α, IL1-β, IL-10, IL-4, IL-5, TGF-β1 and VEGF by Enzyme-Linked Immunosorbent Assay (ELISA) techniques using specific antibodies (purified and biotinylated) and cytokine standards, according to the manufacturer's instructions (R&D Systems, Minneapolis, USA). Optical densities were measured at 450 nm in a microplate reader (μQuant, Biotek Instruments Inc.). Cytokine levels were expressed in pg, sensitivities were >10 pg/ml.

PGE_2_, PGD_2_ and LTB_4_ were measured using ELISA according to the manufacturer's instructions (BD Biosciences and Cayman Chemical). The optical density of samples was determined at 420 nm in a microplate reader (μQuant, Biotek Instruments Inc.), and concentrations of eicosanoids were calculated based on the standard curve. The detection limit was 7.8 pg/ml for PGE_2_, 19.5 pg/ml for PGD_2_ and 3.9 pg/ml for LTB_4_.

### Total protein quantification

Total proteins were quantified in a homogenate fluid obtained from tissue sections of wounds treated with vehicle-control or 16 U HYAL by Coomassie protein assay reagent (Rockford, USA), according to the manufacturer's instructions.

### Measurement of myeloperoxidase (MPO)

To determine the accumulation of neutrophils, MPO activity was assayed in wounds lysate according previous reports [24], [32] with minor modifications as described in the Protocol S1.

### Measurement of hydroxyproline

The amount of hydroxyproline present in the biopsies, which represents the collagen content in the wounds, was determined as previously described [33] with minor modifications as described in the Protocol S1.

### Total RNA Extraction and qRT-PCR

To verify the expression of mRNA for PPAR α, PPAR γ and PPAR δ, the RNA was extracted from wounds biopsies after 16 U HYAL treatment and were analyzed by qRT-PCR. Relative quantification was performed using the ΔΔCt method according to the Protocol S1.

### Statistical analysis

Statistical analyses were performed using GraphPad software (San Diego, CA, 176 USA). Data were expressed as mean ± standard error of mean (SEM). Statistical variations among groups were determined using one way analysis of variance (ANOVA) followed by Dunnett's post-test or two-way- ANOVA when appropriated. Values of p<0.05 were considered significant.

## Results

### HYAL increased migration and proliferation of fibroblasts *in vitro* in a dose-dependent manner

The activity of HYAL (0.1 to 32 U) and its influence on proliferation and/or migration of 3T3 mouse fibroblasts were investigated using the scratch assay. HYAL dose-dependently enhanced the cell number in the gap with values ranging from 20±3.8% (0.1 U) to 92±6.3% (16 U). PDGF was used as positive control and exhibited a stimulatory effect of 52±4.7% (2 ng/ml) (Figure S1). Increased cell numbers can be due to immigration and/or proliferation of the migrated cells. To better distinguish between these two effects, mitomycin C (5 µg/ml) was added to the “wounded” monolayer cultures of fibroblasts together with either PDGF (2 ng/ml) or HYAL (0.1 U to 32 U). As addition of mitomycin C prevents mitosis and thereby proliferation, the remaining increase in the cell number is only due to migration [34], [35]. The total cell numbers slightly decreased using mitomycin C and either PDGF or HYAL for all tested concentrations (see Figure S1) indicating that the observed effect in the *in vitro* scratch assay may be mainly due to migration and only marginally to proliferation.

### HYAL accelerates wound closure *in vivo*

**Figure 1:**
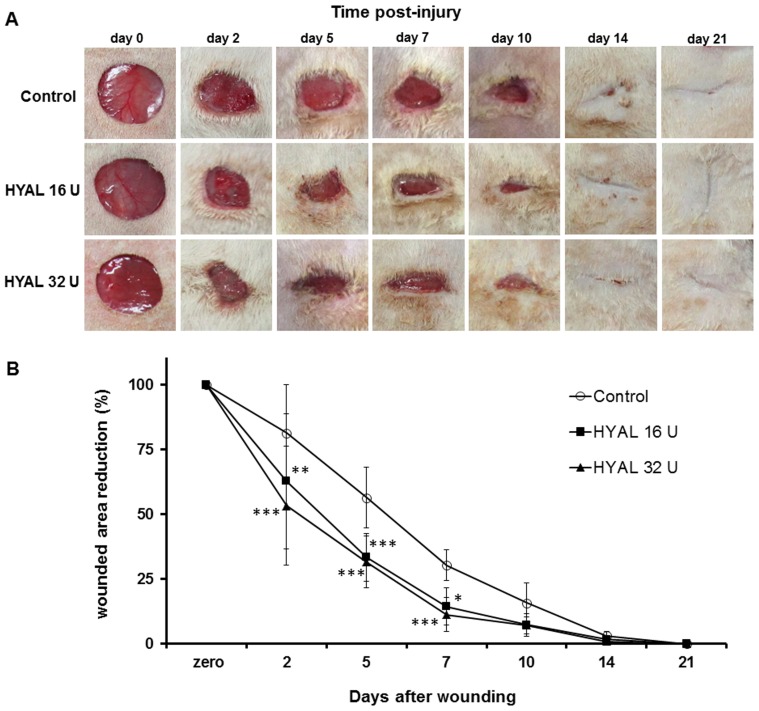
Topical application of HYAL accelerates wound closure in full-thickness excisional wounds. (A) Representative photographs taken from the 150 mm diameter full-thickness wounds of the Wistar rats. Macroscopic changes in skin wound sites induced by topical application of vehicle, HYAL 16 U and HYAL 32 U at day 0 (picture taken immediately after injury) 2, 5, 7, 10, 14 and day 21 are shown. (B) Rate of wound closure induced by topical application of vehicle (control group), HYAL 16 U and HYAL 32 U at day 2, 5, 7, 10, 14 and day 21 are given. Data are expressed as percentage of reduction area from the original wound size (day zero). Values are mean ± SEM (n = 8 to 16 wounds/group), *P<0.05, **P<0.01, ***P<0.001 compared to control group by two-way-ANOVA.

As showed in Figure 1, the wounds treated with 16 U or 32 U HYAL presented faster and higher reepithelialization compared to vehicle-control group. Selected doses of 16 U and 32 U were based on the *in vitro* scratch assay, in which these concentrations exhibited the maximum stimulatory effects on fibroblasts proliferation and migration, as well as on our previous *in vivo* studies using HYAL [20]. Wound closure area observed in the 16 U and 32 U HYAL reached 38% and 46% compared to only 19% in the controls already in the early stage (2^nd^ day), respectively. After 5 and 7 days the closure wound in the 16 U and 32 U HYAL reached 67% and 68% (at day 5) and 85% and 89% (at day 7) as compare to 40% and 69% observed in control group, respectively. Interesting, by day 14 the wounds of HYAL-treated rats were completely closed, whereas the wounds of control rats had not yet completely healed. This observation suggests that HYAL positively influenced the wound healing process and that 16 U or 32 U HYAL treatments healed the full thickness wound in a similar speed. Therefore, the following experiments were performed using only 16 U HYAL.

### HYAL affects cellular recruitment and edema formation

**Figure 2:**
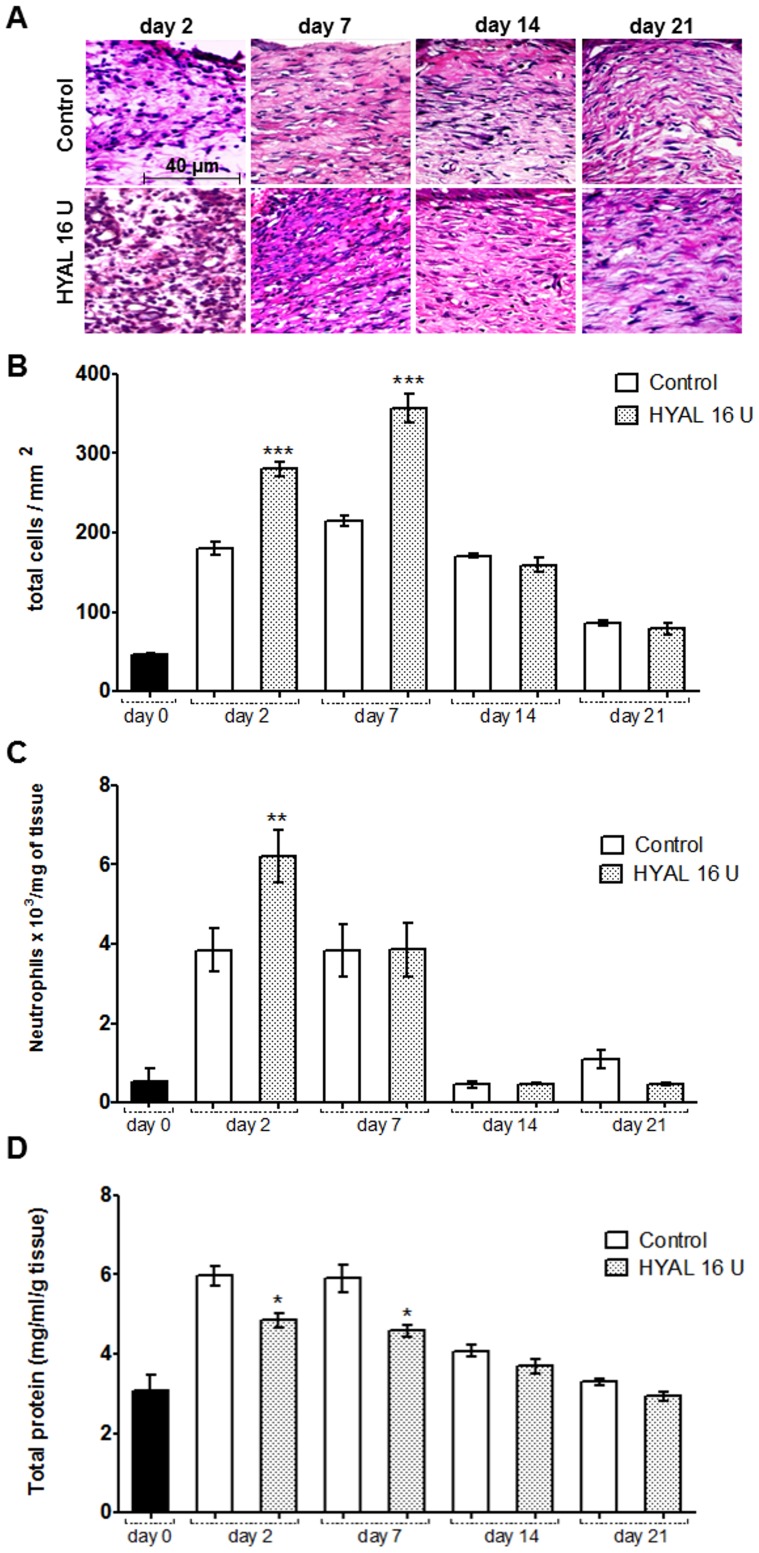
HYAL affects cellular recruitment and edema formation. Animals were topically treated either with vehicle (control group) or HYAL 16 U daily. Paraffin-wound sections were stained with HE to evaluate the inflammatory infiltrate response by image analysis. (A) The sections were photographed at 400x. The ImageJ software was used to count the inflammatory cells in wound tissue specimens at day 2, 7, 14 and 21 post wounding in at least ten random optic fields per group. (B) Histogram of a quantitative analysis of inflammatory infiltrate counted. (C) Tissue neutrophil accumulation determined by MPO levels in wound biopsies. (D) The total protein content was measured according to Coomassie assay. Values represent mean ± SEM (n = 8 wounds/group), *P<0.05, **P<0.01, ***P<0.001 compared to control group by one-way-ANOVA.

Histological analysis from the skin revealed significantly increased cellularity in the wounds of 16 U HYAL treated rats at days 2 and 7 compared to controls (Figure 2A, B). The density of cells 14 and 21 days post wounding were similar between treated and control groups and gradually decreased to physiological levels at 21^st^ day. To evaluate whether neutrophils may have accumulated or have been activated, the activity of myeloperoxidase (MPO) was studied. As expected, MPO activity was very low in the intact skin (day zero) (Figure 2C). However, at day 2 post-wounding, MPO levels markedly increased in the animals treated with 16 U HYAL. At day 7, MPO activity in treated rats declined to similar levels as in the controls and both gradually declined thereafter to normal concentration at day 14 as observed in the day zero. To study if the decrease in MPO activity may have an influence on the edema we analyzed the total protein content in homogenate skin biopsies. We observed that HYAL significantly reduced the edema formation evidenced by lower amount of protein at day 2 and at day 7 after excision wounds being daily treated with 16 U HYAL and compared to vehicle-control group (Figure 2D). After 14 and 21 days no difference was observed between experimental groups, and the amount of protein declined to similar concentrations detected in unwound control tissue.

### HYAL induces cytokine release and eicosanoid generation, and temporarily increases TGF-β in the skin wound biopsies

**Figure 3:**
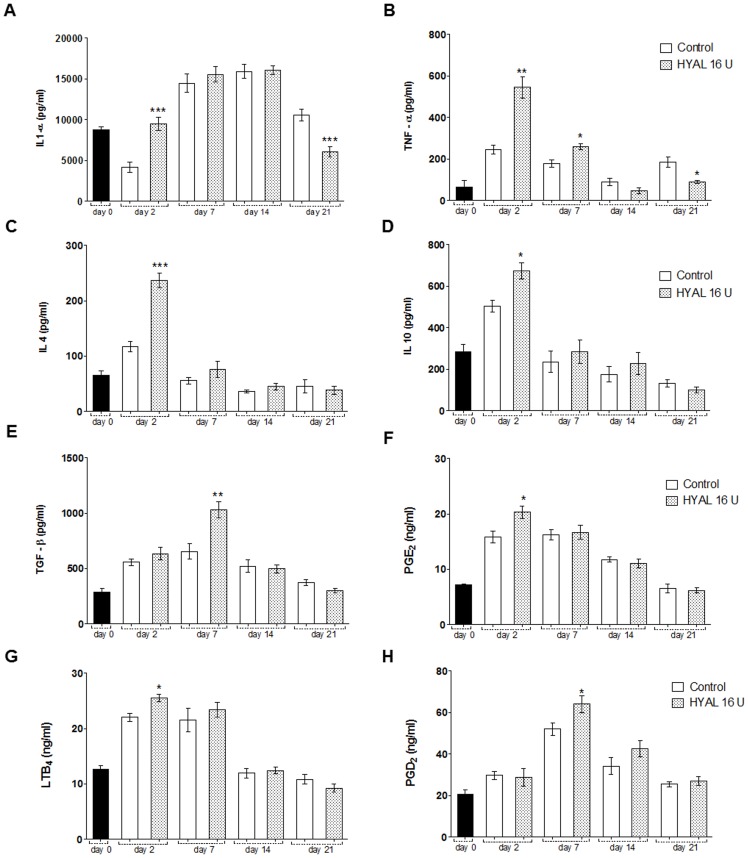
HYAL modulates cytokines and induces eicosanoid generation in the skin wound biopsies. Homogenates were prepared from the wound biopsies obtained from animals at day 0, 2, 7, 14 and 21 treated with 16 U HYAL or with vehicle-control. (A) IL1-α, (B) TNF-α, (C) IL-4, (D) IL-10, (E) TGF-β, (F) PGE_2_, (G) LTB_4_ and (H) PGD_2_ were assayed by ELISA. Data are means ± SEM (n = 8 wounds/group), *P<0.05, **P<0.01, ***P<0.001 compared to control group by one-way-ANOVA.

Neutrophils were proven to be attracted to the wounded sites which may be induced by various cytokines. However, neutrophils themselves can also be activated releasing cytokines which have an impact on further steps in the wound healing process. Therefore, we evaluated the impact of HYAL on different cytokines. In fact, 16 U HYAL altered the release of pro- and anti-inflammatory cytokines in a time-dependent manner (Figure 3). Enhanced production of IL1-α, TNF-α, IL-4 and IL-10 at day 2 was observed compared to vehicle-control group (Figure 3A, B, C e D). Except of IL1-α all other cytokines declined at day 7 and this continuously. Only TNF-α was still slightly, but significantly increased at day 7 compared to the controls. After 14 days, there were no significant changes in the levels of the studied cytokines, while at day 21, a significant decrease in the IL1-α and TNF-α amount could be observed. TGF-β production was increased after 2 days, peaked at day 7 and then decreased after 14 and 21 days to control level in the wound biopsies (Figure 3E). Levels of IL-6, IL1-β, IFN-γ and IL-5 were not detected after HYAL treatment during the time-course of this experiment. Eicosanoids were also altered by HYAL treatment. Compared to controls, we observed a significant peak in the production of PGE_2_ and LTB_4_ after 2 days of HYAL treatment (Figure 3F, G). Subsequently, both lipid mediators decreased to baseline levels, after 14 days for LTB_4_ and 21 days for PGE_2_ post-wounding, respectively. Low concentrations of PGD_2_ where observed in the first days of HYAL treatment, however a significant increase in the production of PGD_2_ was observed at day 7 (Figure 3H).

### HYAL temporarily affects collagen accumulation

**Figure 4:**
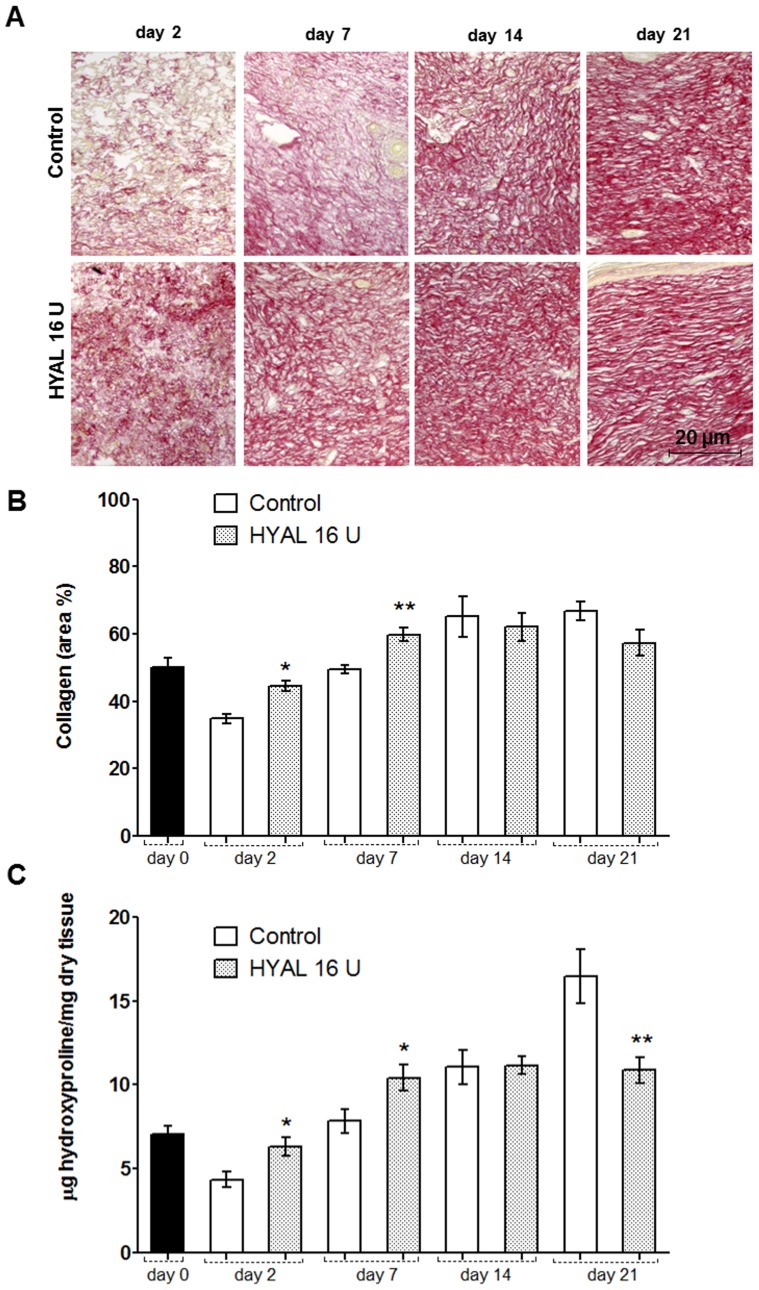
Collagen accumulation in wound areas of HYAL and vehicle-control treated rats at day 2, 7, 14 and 21 after wounding. (A) Representative photomicrograph of wounds tissue sections stained with picrosirius red staining (200x), note the collagen intensity and disposition of fibers (red). (B) Collagen content measured by digital densitometry is shown as a result of collagen content in each specimen in percentage. (C) Determination of wound hydroxyproline content as an indicator of collagen levels. µg of hydroxyproline/mg of dry wound specimen content was measured at day zero, 2, 7, 14 and 21 after injury. Data represent means ± SEM (n = 8 wounds/group), *P<0.05, **P<0.01 compared to control group by one-way-ANOVA.

Collagen deposition is an important event in the development of granulation tissue. We could observe that HYAL treated wounds revealed a marked and robust increase in the organization of collagen fibers, detected after staining with Picrosirius red in the wound biopsy, bridging the gaps in the skin compared to the vehicle-treated animals (Figure 4A). At days 2 and 7, the collagen content in the 16 U HYAL treated group was significantly higher than in the vehicle-control group, whereas after 14 and 21 days the collagen amount was similar between the groups with a slight decrease in the HYAL treated group at day 21 post wounding (Figure 4B). To confirm these histological findings, the collagen content was also measured by calculating the amount of hydroxyproline in the homogenate tissue of the wounds after 2, 7, 14 and 21 days post-wounding. The results of the hydroxyproline content in the skin after 2 days and 7 days by daily 16 U HYAL treatment showed a significant higher concentration compared to the controls (Figure 4C). Interestingly, after 21 days of 16 U HYAL wound treatment, the collagen content was significantly reduced compared to the control group.

### HYAL affects neovascularization

**Figure 5:**
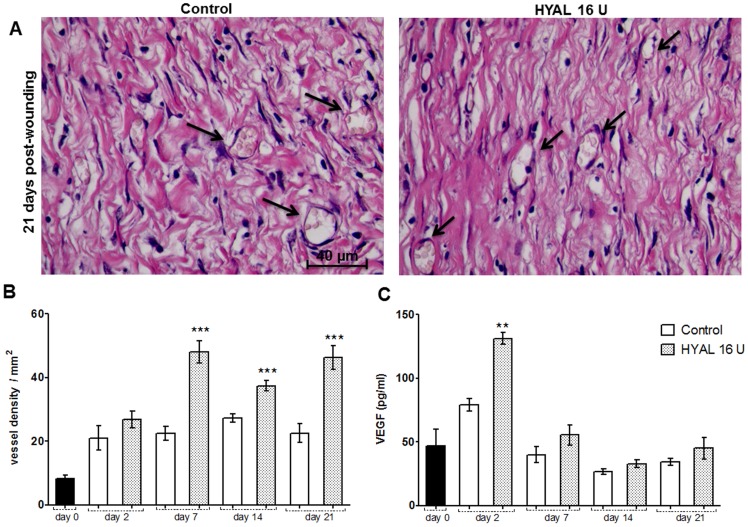
Neovascularization induced by HYAL. Animals were topically treated either with vehicle (control group) or HYAL 16 U daily. Paraffin-wound sections were stained with HE to evaluate the angiogenic response by image analysis. The sections were photographed at 400x. The ImageJ software was used to count the blood vessels in wound tissue specimens at day 2, 7, 14 and 21 post wounding in at least ten random optic fields per group. (A) Representative photomicrograph of blood vessels (black arrow) in wound tissue specimens at day 21 post-wounding. (B) Histogram of a quantitative analysis of vascular density counted. (C) Vascular endothelial growth factor (VEGF) expression measured in the supernatant of wound tissue homogenate by ELISA. Data represent means ± SEM (n = 8 wounds/group), **P<0.01, ***P<0.001 compared to control group by one-way-ANOVA.

The number of blood vessels in the HYAL treated wound was determined using different techniques. Figure 5A exemplarily illustrates photomicrographs of blood vessels in tissue sections from the wounds. The number of blood vessels in the 16 U HYAL treated wounds determined by morphometric analyses was increased after 7, 14 and 21 days (Figure 5B). Levels of VEGF, a signal protein that stimulates angiogenesis, measured by ELISA in the wound tissue homogenate was enhanced in 16 U HYAL treated wounds at day 2 post wounding (Figure 5C).

### HYAL differently influenced PPAR gene expression in the wound tissue

**Figure 6:**
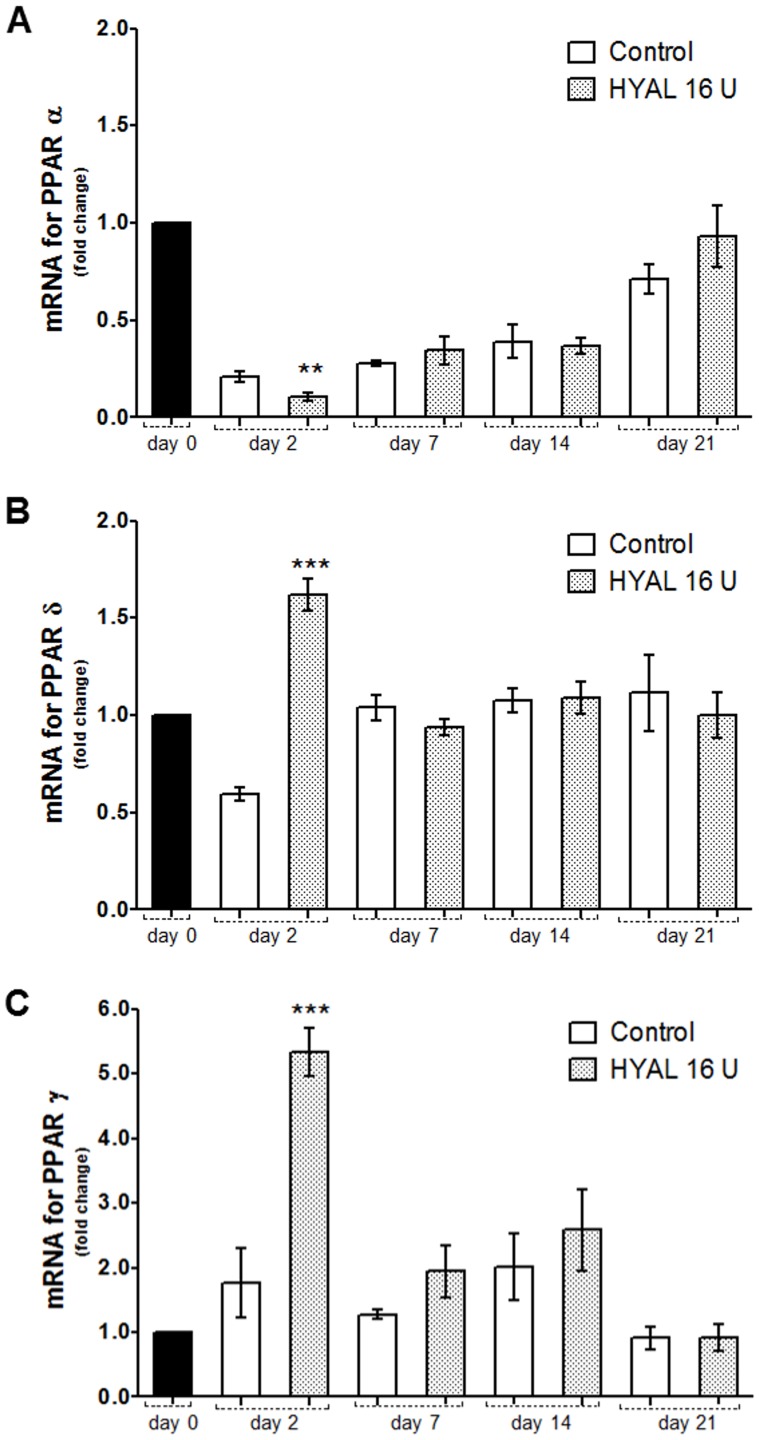
PPARs gene expression in skin biopsies dissected from the cutaneous wounds after HYAL treatment analyzed by qRT-PCR. A) mRNA for PPAR α, B) PPAR δ and C) PPAR γ determined by qRT-PCR. *Ppar* expression was normalized by *Gapdh* and *beta-actin*. Animals were topically treated with the vehicle (control group) or 16 U HYAL daily for 2, 7, 14 and 21 days, respectively. Data represent means ± SEM (n = 8 wounds/group), **P<0.01, ***P<0.001 compared to control group by one-way-ANOVA.

PPAR α and δ (also called β) have been shown to be important for the rapid epithelialization of wound skin and PPAR γ during the resolution phase of wound repair. To study the impact of HYAL on PPAR gene expression (*Ppara*, *Pparg* and *Ppard*), the skin extracts dissected from the cutaneous wounds were analyzed by qRT-PCR. The three PPAR isotypes were expressed in the skin at all-time points, but in a different manner (Figure 6). mRNA for PPAR α (*Ppara*) was inhibited in the cutaneous wound, compared to unwounded skin (day 0). Interestingly, a time-dependent increased expression of *Ppara* was observed after daily treatment of 16 U HYAL until day 21, reaching finally a similar expression level as the physiological one (Figure 6A). Levels of PPAR δ mRNA were only increased at day 2 (fold increase 1.62), but returned to levels measured in the unwounded tissue (Figure 6B). mRNA of PPAR γ showed the highest fold increase at day 2 (fold increase 5.3) and decreased to levels in the range of the controls (Figure 6C).

## Discussion

Wounds cause discomfort and are prone to infection and other complications. Moreover, diseases like diabetes, immunosuppression diseases, ischemia and ageing lead to a delay in wound healing. Therefore, agents that accelerate healing are important and research to find potent agents are a challenging task. Our aim was to evaluate whether HYAL is not only a spreading agent to improve the bioavailability of drugs as known from the literature [17], [18] but has also a wound healing potential.

The *in vitro* scratch assay performed with HYAL and 3T3 mouse fibroblasts provided first preliminary insights that HYAL mainly influence the migration of the fibroblasts. It can be assumed that then also the rebuilding of new granulation tissue may be positively influenced [3]. This *in vitro* assay has been demonstrated to be a convenient and suitable in vitro test that gives robust and reproducible results for the migration of fibroblasts in an artificial wounded area [23], [36], [37]. However, it has to be taken into account that the 3T3 fibroblast cell line may be behave differently from primary fibroblasts and most important, this *in vitro* assay cannot replace *in vivo* studies. Therefore, we studied the wound healing activity of HYAL in the *in vivo* model of the skin excisional wound in rats. Macroscopic examination showed that the healing rate of HYAL treated wounds was significantly higher already in the early phase at day 2 compared to the controls (Figure 1A). The wound closure involves a complex orchestrated interaction of different cell types including neutrophils, macrophages, keratinocytes, fibroblasts and endothelial [6]. It is well known that recruitment of neutrophils within the first hours and days and macrophages in the later phase of inflammation that occurs in site of the skin injury is crucial. These cells have the competence to remove cellular debris and dead cells and therefore supporting the wound healing processes [4], [5]. We demonstrate that HYAL increased myeloperoxidase activity in the neutrophils at day 2 which is probably related to an enhanced recruitment of mononuclear cells as reported in the literature [32]. Moreover, at 2^nd^ and 7^th^ day post-wounding cellularity and release of cytokines, growth factors and lipid mediators is increased according to our data (Figure 2 and 3). Pro-inflammatory cytokines, including IL1-α, IL1-β, IL6, and TNF-α, play an important role in wound repair. They influence various processes at the wound site, such as stimulation of keratinocyte and fibroblast proliferation, synthesis and breakdown of extracellular matrix proteins, fibroblast chemotaxis, and regulation of the immune response [6]. In addition, IL-10 plays a major role in the limitation and termination of the inflammatory response, regulating the growth and differentiation of various immune cells, keratinocytes and endothelial cells [5]. The observed effect suggested that HYAL has a different impact on the release of the cytokines depending on the stage of the inflammatory process and could therefore control the degree and duration of the inflammatory response. Beside cytokines, lipid mediators such as prostaglandins (PGs) and leukotrienes (LTs) can also be released during different stages of the healing phases. They are reported to play a crucial role in the initiating and resolution of acute inflammation during wound healing [38]–[40]. Our findings (Figure 3F, G, H) corroborate with previous data [41], [42], demonstrating that during the wound repair, a gradual shift in the metabolism of arachidonic acid from the pro-inflammatory PGE_2_ to anti-inflammatory PGD_2_ occurs and that this shift in the metabolism of arachidonic acid may be responsible for initiating endogenous mechanism resulting in wound healing.

We could also demonstrate that HYAL differently affects the expression of transcription factors peroxisome proliferator-activated receptors (PPARs), which have received attention because of their protective and healing properties in tissue injury and wound repair [9], [43], [44]. Our results showed increased levels of PPAR δ and PPAR γ mRNA, but decreased levels of PPAR α mRNA after HYAL treatment in the first stage (day 2) compared to the unwounded tissue and the vehicle-control in the wounded tissue (Figure 6). The increased gene expression corroborated with previous reports where PPAR δ was demonstrated to be activated by the stress-associated protein kinase pathway in response to inflammatory cytokines, such as TNF- α and IL1, and down-regulated by TGF-β1 after a skin injury, playing an important role for new tissue development [8], [45], [46]. mRNA increased expression of PPAR γ accompanied with increased levels of PGD_2_ is in agreement with the literature [41]. It is reported that upregulation of PPAR γ during the resolution phase of wound repair concomitant with PGD_2_ expression is responsible for initiating the endogenous mechanism which results in healing/resolution.

Collagen is the major component of the connective tissue. Hence, the healing processes depend on its regulatory production, deposition and subsequent maturation. As the ECM can have positive or negative effects, the right balance and shifts in synthesis versus catabolism of collagen during the healing process are important to prevent and avoid keloid scarring. Keloid is often considered to be the result of a prolonged proliferative and a delayed remodeling phase which contain disorganized and large collagen fibers, whereas hypertrophic scars exhibit thin fibers which are organized into nodules [7], [47], [48]. The enhanced collagen in skin wounds of HYAL treated rats in the first stages stabilizes the new granulation tissue formation and may consequently accelerate the rate of wound closure. Importantly, collagen amount was diminished at day 21 post-wounding which may contribute to anti-fibrotic scar formation and a better distribution and organization of collagen fibers (Figure 4). During the entire process of healing, modulation of the ECM metabolism has been extensively demonstrated to be mainly regulated by adequate temporal secretion of TGF-β1 [49]. At early stages the synthesis and deposition of collagen are essential and treatment of wounds with anti TGF-β antibodies resulted in delayed wound healing, demonstrating the importance of collagen content at this stage for proper healing [50]. Therefore, the observed increased TGF-β1 secretion at day 7 after HYAL treatment (Figure 3E) is in agreement with this previous report. Altogether, these observed effects of HYAL on collagen content and maturation process in rat skin may be beneficial for clinical use helping to prevent exacerbated wound healing processes such as hypertrophic and contracted scars.

Another important fact during wound healing is the angiogenesis that exerts dual function by providing the essential nutrients required and the oxygen to the wounded site, thus promoting granulation tissue formation [1], [2]. HYAL treatment was found to increase the angiogenesis as evidenced histologically by the increased blood vessel density in the wound (Figure 5) and the expression of VEGF, which might be responsible to trigger the angiogenesis. The observed decrease in the VEGF protein expression after 2 days is in agreement with the previous studies showing that transcription and secretion are elevated in the acute wounds. Expression of VEGF proteins during healing of cutaneous wounds was demonstrated to be induced within 24 h of skin injury, was maximal at 2–3 days and declined to basal level after 7 days of skin injury [51], [52]. Moreover, it has been suggested that low molecular weight HA stimulates formation on new blood vessels and contributes to wound healing [53]–[55]. Therefore the angiogenic activity may be attributed to degradation products of hyaluronic acid of a specific size by HYAL, which has yet to be proven experimentally.

## Conclusions

Taken together, under *in vivo* conditions we could show that HYAL accelerates the wound closure in the full-thickness excisional model in Wistar rats and give further insights how this wound healing properties can be explained on the molecular level. HYAL regulates the inflammatory response by mediating pro and anti-inflammatory cytokines like TNFα, IL-1α, IL-10 and IL-4, lipid mediators like PGE_2_, LTB_4_ and PGD_2_, and the transcription factors PPARs δ, α and γ. Moreover, the enzyme contributes to the balance between synthesis and deposition of collagen and promotes angiogenesis. Therefore, HYAL may have a potential as a healing promoting agent for cutaneous injuries.

## Supporting Information

Figure S1 Effect of HYAL on the migratory and proliferative activities of 3T3 mouse fibroblasts in the scratch assay. The experiments were performed in the absence (open bars) or presence (filled bars) of 5 µg/ml of antimitotic mitomycin C after 14 h incubation (37°C, 5% CO_2_) in DMEM medium supplemented with 10% fetal bovine serum. HYAL was tested at concentration ranging from 0.1 U to 32 U. PDGF-BB was used as positive control at 2 ng/ml concentration. Data are expressed as percentage of cell numbers in the injured area, compared to the control group (DMEM medium only). Bars represent the mean ± SEM of three independent experiments, **P<0.01, ***P<0.001 compared to control group by two-way-ANOVA.
(TIF)

Table S1 Comparison of hyaluronidase enzyme activity in solution with in the gel preparations.
(DOC)

Protocol S1 (DOC)
