# Supplementary figures and images for: Hyaluronidase Modulates Inflammatory Response and Accelerates the Cutaneous Wound Healing
Source: PLoS One. 2014 Nov 13;9(11):e112297. doi: 10.1371/journal.pone.0112297 (PMC4230982; doi:10.1371/journal.pone.0112297)

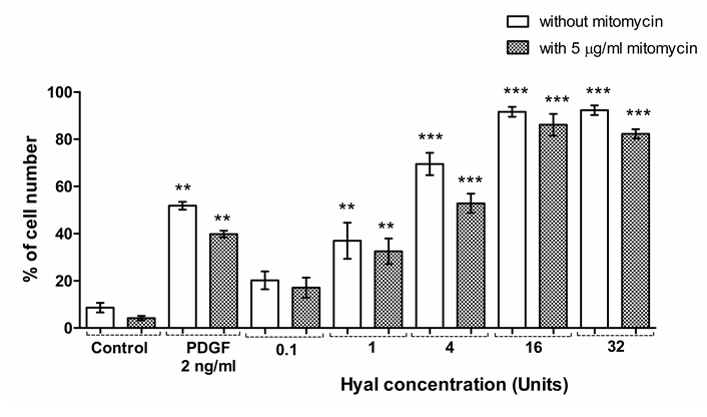

Supplement: Figure S1 — Effect of HYAL on the migratory and proliferative activities of 3T3 mouse fibroblasts in the scratch assay. The experiments were performed in the absence (open bars) or presence (filled bars) of 5 µg/ml of antimitotic mitomycin C after 14 h incubation (37°C, 5% CO2) in DMEM medium supplemented with 10% fetal bovine serum. HYAL was tested at concentration ranging from 0.1 U to 32 U. PDGF-BB was used as positive control at 2 ng/ml concentration. Data are expressed as percentage of cell numbers in the injured area, compared to the control group (DMEM medium only). Bars represent the mean ± SEM of three independent experiments, **P<0.01, ***P<0.001 compared to control group by two-way-ANOVA. (TIF) [file pone.0112297.s001.tif]
