# Supplementary material for: Hyaluronidase Modulates Inflammatory Response and Accelerates the Cutaneous Wound Healing
Source: PLoS One. 2014 Nov 13;9(11):e112297. doi: 10.1371/journal.pone.0112297 (PMC4230982; doi:10.1371/journal.pone.0112297)
Supplement: Table S1 — Comparison of hyaluronidase enzyme activity in solution with in the gel preparations. (DOC) [file pone.0112297.s002.doc]

***Table S1****. Comparison of hyaluronidase enzyme activity in solution with in the gel preparations.*

|  | **Hydrolytic Activity (%)** | | | |
| --- | --- | --- | --- | --- |
|  | **16 U/0.2 g gel** | | **32 U/0.2 g gel** | |
| **TRU** | **S** | **G+** | **S** | **G+** |
| 1.0 | 63.7 ± 8.4 | 56.8 ± 11.2 | 75.5 ± 5.1 | 66.6 ± 5.6 |
| 2.0 | 94.7 ± 3.5 | 82.2 ± 7.3 | 94.0 ± 7.8 | 86.9 ± 4.1 |

*TRU: turbidity reducing activity; S: solution;* G+*: gel plus hyaluronidase.*
