# Supplementary material for: Hyaluronidase Modulates Inflammatory Response and Accelerates the Cutaneous Wound Healing
Source: PLoS One. 2014 Nov 13;9(11):e112297. doi: 10.1371/journal.pone.0112297 (PMC4230982; doi:10.1371/journal.pone.0112297)
Supplement: Protocol S1 — (DOC) [file pone.0112297.s003.doc]

**SUPPORTING INFORMATION – PROTOCOL S1**

**Hyaluronidase modulates inflammatory response and accelerates the cutaneous wound healing**

Marcio Fronza^1,4^, Guilherme F. Caetano^2^, Marcel N. Leite^2^, Claudia S. Bitencourt^1^, Francisco W.G.Paula-Silva^1^, Thiago A.M. Andrade^2^, Marco A.C. Frade^2^, Irmgard Merfort^3^, Lúcia H. Faccioli^1^

^1^Departamento de Análises Clínicas, Toxicológicas e Bromatológicas, Faculdade de Ciências Farmacêuticas de Ribeirão Preto, Universidade de São Paulo, Ribeirão Preto, São Paulo, Brazil.

^2^Departamento de Clínica Médica, Divisão de Dermatologia, Faculdade de Medicina de Ribeirão Preto, Universidade de São Paulo, Ribeirão Preto, São Paulo, Brazil.

^3^Department of Pharmaceutical Biology and Biotechnology, University of Freiburg, Freiburg, Germany.

^4^ Departamento de Farmácia, Universidade de Vila Velha, Vila Velha, Espirito Santo, Brazil.

**METHODS**

*In vitro cell migration assay*

The proliferation and migration abilities of fibroblasts exposed to hyaluronidase were assessed using a scratch wound assay which measures the expansion of a cell population on surfaces . Swiss 3T3 albino mouse fibroblasts (4×10^5^ cells/mL) were cultured with DMEM medium containing 10% FBS in 24-well plates containing glass coverslips previously treated with collagen type I (40µg/ml, 2 hours at 37°C) to nearly confluent cell monolayers. An artificial linear wound (scratch) was then carefully introduced using 200µL sterile pipette tips. The coverslips were rinsed with sterile saline (PBS) in order to remove the cellular debris. After, the fibroblasts monolayers were divided into groups: DMEM medium only (control group), platelet derived growth factor (2 ng/ml) (positive control) and HYAL (0.1 to 32 U) was added to a set of 4 coverslips per dose and incubated for 14 h at 37ºC with 5% CO_2_. After treatment, the cells were fixed and stained with DAPI. Pictures of scratched areas were made using camera Leica (Leica Microsystems Ltd, Heebrugg, Switzerland) coupled to Leica microscope (Leica Microsystems GmbH, Wetzlar, Germany) and connected to a computer where the images were processed and stored. The cellular migration into the wounded area was quantified using CellC software and the results were expressed as percentage of cells that proliferate and/or migrate into the injured area after being treated with different concentrations of hyaluronidase in comparison to the control group.

*Assay of hyaluronidase enzyme activity*

The enzymatic activity of the HYAL was determined before and after the gel preparations using the methodology described by Pessini *et al.* (2001) . Briefly, the assay mixture contained acetate buffer 0.2 M pH 6.0 (containing 0.15 M NaCl), 50 mg hyaluronan (0.5 mg/ml in acetate buffer) and the enzyme (or gel preparations) in a final volume of 0.5 ml was incubated for 15 min at 37 ºC. After incubation, the reaction was stopped with 1 ml of 2.5% (w/v) acetyltrimethylammonium bromide in 2% (w/v) NaOH. After 10 min, the absorbance was read at 400 nm against the blank reaction control prepared in the same conditions without hyaluronan. Turbidity reducing activity was expressed as a percentage of the remaining hyaluronan, taking the absorbance of a tube in which no enzyme was added as 100%. Turbidity reducing units were expressed as the quantity of HYAL necessary to hydrolyze 50% (25 mg) of hyaluronan.

*Evaluation of inflammatory infiltrate and angiogenesis by image analysis*

Paraffin-wound sections were stained with hematoxylin-eosin (HE) to evaluate the inflammatory infiltrate response by image analysis. The sections were photographed in a blinded fashion at 400x using Leica DM 4000B microscope equipped with a Leica DFC 280 camera (Leica Microsystems, Germany) and Leica Application Suite Version 3.2.0 software. Ten different fields (5 from the superficial dermis, 2 from the deep dermis, and 3 from a neo-issue area) were examined and a region of interest (ROI) of 900 x 300 pixels was acquired for each field. The ImageJ 1.48 software (U.S. National Institutes of Health, USA) was used to count the inflammatory cells and blood vessels in each field. Four different mice were used for each treatment at each time (n=8 wounds/group) and the data are reported as the average number of inflammatory cells and blood vessels per group .

*Evaluation of collagenesis by imaging*

Quantification of collagenesis was measured in Sirius red-stained sections as follow: ten different fields were photographed at 200X and a ROI of 320 x 40 pixels (= 2.62 x 0.33 μm, converted using ImageJ) was selected for each field. The distribution of red color (collagen) was quantified as a percentage of the total number of pixels in the ROI using the “Threshold color” plug-in of the ImageJ software. Four different mice were used for each treatment at each time (n=8 wounds/group) and the final results are reported as the average distribution of collagen per treatment .

*Biochemical measurement of myeloperoxidase (MPO)*

A portion of the skin biopsy was weighed and lysed with 0.2% NaCl solution (15 ml/mg of tissue) for 30s followed by the addition of 1.6% NaCl containing 5% glucose and centrifuged 5 minutes at 3000 *g*. The cell pellet was resuspended in 0.05 M NaPO_4_ buffer, pH 5.4, containing 0.5% hexadecyltrimethylammonium bromide and re-homogenized. Aliquots of 1.0 ml of the suspension were transferred to Eppendorf tubes followed by 3 cycles of freezing-thawing using liquid nitrogen. These aliquots were centrifuged for 15 min at 5000 *g*. The assay was performed in 96-well plates. 5.0 μl of the supernatants diluted in 45.0 μl 0.08 M NaPO_4_ were added to each well and the MPO activity was detected spectrophotometrically at 450 n after adding 25 μl 1.6 mM tetramethylbenzidine followed by 100 μl 0.5 mM H_2_O_2_. The results were reported as the total number of neutrophils x 10^3^/mg tissue by comparing the absorbance of the tissue supernatant to a standard curve generated using mouse peritoneal neutrophils .

*Measurement of hydroxyproline content in the wounds*

Approximately 100 mg of each wound biopsies taken on day zero and at 2^nd^, 7^th^, 14^th^ and 21^st^ day post-wounding were dried at 60°C overnight and weighed again. Dry tissue was hydrolyzed in 6M HCl (100 μl HCl /1.0 mg of dry tissue) for 4h at 110 °C. 500 μl of each hydrolysate sample was neutralized with 6M NaOH. Then, 10 μl of the test samples and standard solutions prepared with hydroxyproline were added to a 96-well microplate. Ninety microliters of 0.056M chloramine T solution was added to each sample and incubated for 25 minutes at room temperature. After, 100 μl 1M Ehrlich’s reagent was added to the oxidized samples and the microplate incubated for 15 minutes at 60 ºC. After incubation, the microplate was cooled for 10 minutes, homogenized and the absorbance was measured at 550 nm in a microplate reader. The concentrations of hydroxyproline in tissue homogenates were determined per volume of HCl used and finally per milligram of dry tissue, based on a standard curve .

*Total RNA Extraction and qRT-PCR*

To evaluated the expression of mRNA for PPAR α, PPAR γ and PPAR δ, the RNA from the wounds biopsies was extracted using the Illustra RNAspin Mini Isolation Kit (GE Healthcare, Buckinghamshire, UK) and samples were treated with DNAse, according to manufacturer protocol. The RNA quantification and purity was determined using NanoDrop 1000 (Thermo Fisher Scientific Inc., Wilmington, EUA) at 230, 260 and 280 nm wavelengths. cDNA (complimentary DNA) was synthesized from 500 ng of total RNA using random primers (High Quality cDNA Reverse Transcriptase Kit). Aliquots of 2 µl of the total cDNA were amplified by qRT-PCR using the primers for *ppara* (Rn00566193), *pparg* (Rn00440945) and *ppard* (Rn00565707) (TaqMan Gene Expression Assay, Applied Biosystems). *Gapdh* (4352338E) and *beta-actin* (4352340E) were used as internal controls. Amplification was performed under the following conditions: denaturation at 95 °C for 10 min; followed by 40 cycles of 95 °C, 15 s and 60 °C, 1 min. Relative quantification was performed using the ΔΔCt Method.

**REFERENCES**

1. Fronza M, Heinzmann B, Hamburger M, Laufer S, Merfort I (2009) Determination of the wound healing effect of Calendula extracts using the scratch assay with 3T3 fibroblasts. Journal of Ethnopharmacology 126: 463-467.

2. Pessini AC, Takao TT, Cavalheiro EC, Vichnewski W, Sampaio SV, et al. (2001) A hyaluronidase from Tityus serrulatus scorpion venom: isolation, characterization and inhibition by flavonoids. Toxicon : official journal of the International Society on Toxinology 39: 1495-1504.

3. Noursadeghi M, Tsang J, Haustein T, Miller RF, Chain BM, et al. (2008) Quantitative imaging assay for NF-kappaB nuclear translocation in primary human macrophages. Journal of immunological methods 329: 194-200.

4. Andrade TA, Iyer A, Das PK, Foss NT, Garcia SB, et al. (2011) The inflammatory stimulus of a natural latex biomembrane improves healing in mice. Brazilian journal of medical and biological research 44: 1036-1047.

5. Souza DG, Cassali GD, Poole S, Teixeira MM (2001) Effects of inhibition of PDE4 and TNF-alpha on local and remote injuries following ischaemia and reperfusion injury. British Journal of Pharmacology 134: 985-994.

6. Reddy GK, Enwemeka CS (1996) A simplified method for the analysis of hydroxyproline in biological tissues. Clinical biochemistry 29: 225-229.
